# The Protective Role of Molecular Hydrogen in Ischemia/Reperfusion Injury

**DOI:** 10.3390/ijms25147884

**Published:** 2024-07-18

**Authors:** Branislav Kura, Jan Slezak

**Affiliations:** Centre of Experimental Medicine, Slovak Academy of Sciences, Dúbravská cesta 9, 841 04 Bratislava, Slovakia; jan.slezak@savba.sk

**Keywords:** apoptosis, inflammation, ischemia/reperfusion, molecular hydrogen, oxidative stress

## Abstract

Ischemia/reperfusion injury (IRI) represents a significant contributor to morbidity and mortality associated with various clinical conditions, including acute coronary syndrome, stroke, and organ transplantation. During ischemia, a profound hypoxic insult develops, resulting in cellular dysfunction and tissue damage. Paradoxically, reperfusion can exacerbate this injury through the generation of reactive oxygen species and the induction of inflammatory cascades. The extensive clinical sequelae of IRI necessitate the development of therapeutic strategies to mitigate its deleterious effects. This has become a cornerstone of ongoing research efforts in both basic and translational science. This review examines the use of molecular hydrogen for IRI in different organs and explores the underlying mechanisms of its action. Molecular hydrogen is a selective antioxidant with anti-inflammatory, cytoprotective, and signal-modulatory properties. It has been shown to be effective at mitigating IRI in different models, including heart failure, cerebral stroke, transplantation, and surgical interventions. Hydrogen reduces IRI via different mechanisms, like the suppression of oxidative stress and inflammation, the enhancement of ATP production, decreasing calcium overload, regulating cell death, etc. Further research is still needed to integrate the use of molecular hydrogen into clinical practice.

## 1. Introduction

Ischemia/reperfusion injury (IRI) refers to the complex pathophysiological sequelae that manifest during reperfusion following a period of tissue ischemia. These sequelae encompass both functional and structural derangements [1]. IRI is associated with a serious clinical manifestations, including myocardial hibernation, acute heart failure, cerebral and gastrointestinal dysfunction, systemic inflammatory response syndrome, and multiple-organ dysfunction syndrome [2]. In the process of ischemia, ATP levels and intracellular pH decrease because of anaerobic metabolism, leading to lactate accumulation. Furthermore, ischemia disrupts cellular homeostasis by compromising the function of ATP-dependent ion channels. This dysfunction leads to the dysregulation of intracellular and mitochondrial Ca^2+^ homeostasis, a phenomenon termed calcium overload [3]. When the blood supply is re-established after prolonged ischemia, local inflammation and reactive oxygen species (ROS) production increase, leading to secondary injury. Cell damage may end with cell death via apoptosis, autophagy, necrosis, and necroptosis [2].

Hydrogen is non-toxic, tasteless, odorless, colorless, highly flammable gas widely distributed in nature and an important component of the body. In the form of gas, it occurs naturally mainly in space. In 2007, Ohsawa et al. discovered that molecular hydrogen could selectively reduce hydroxyl radicals (·OHs) and peroxynitrite (ONOO^−^) in cells, thereby suppressing brain IRI and stroke in a rat model [4]. Subsequent cellular and animal studies, as well as clinical experiments, have demonstrated the preventive and therapeutic effects of molecular hydrogen in various organs, including the brain, heart, lung, pancreas, and liver, through its antioxidant, anti-inflammatory, antiapoptotic, and various other biological effects [5,6,7,8,9]. Several studies have shown that hydrogen could be used to prevent IRI. The authors identified antioxidant, anti-inflammatory, cytoprotective, or calcium-lowering effects as the underlying mechanisms of hydrogen’s mitigating effects on IRI [10,11,12,13].

Administration of hydrogen is well tolerated in humans with no severe adverse effects and considered to be safe despite its flammability and explosiveness [14]. It is well known that hydrogen gas is flammable only at temperatures higher than 527 °C and explodes by a reaction with O_2_ only in the explosive range of hydrogen concentration (4–75%, *v*/*v*) [15]. The most common methods of hydrogen administration include the drinking of hydrogen-rich water and inhalation of hydrogen-enriched gas. Water can be saturated by bubbling with hydrogen gas or chemical reaction with metallic magnesium up to a concentration of 1.6 ppm. Among other delivery methods belong a prebiotic supplementation enriched with hydrogen-producing intestinal bacteria, the injection of hydrogen-rich saline, hydrogen-enriched eye drops, or hydrogen bathing [14].

Hydrogen exhibits a favorable safety profile for clinical applications due to its ability to selectively target detrimental ROS without interfering with essential metabolic redox processes [16]. By virtue of its low molecular weight and substantially higher lipophilicity compared to water (approximately fourfold), hydrogen exhibits exceptional permeability across biological membranes, including those of subcellular organelles. This characteristic confers a significant advantage over other antioxidants [11]. Another great advantage is its zero or low toxicity [17].

In this review, we summarize the most recently published literature concerning the application of molecular hydrogen for IRI in various organs and briefly discuss the potential mechanisms underlying the action of molecular hydrogen in IRI. Actual use of molecular hydrogen in clinical research is also described.

## 2. Mechanisms of Ischemia/Reperfusion Injury

**Figure 1:**
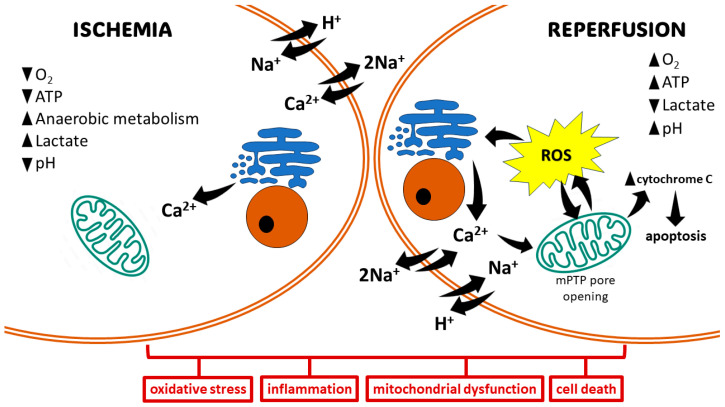
Basic mechanisms of ischemia/reperfusion injury. The ischemic state promotes anaerobic metabolism, resulting in the production of lactate and a drop in intracellular pH. Hypoxia leads to decreased generation of ATP, which in turn affects ion-exchange channels, leading to Na^+^ and Ca^2+^ overload. Mitochondrial damage and electrolyte imbalance in the reperfusion state promote oxidative stress via the opening of the mitochondrial permeability transition pore (mPTP). This induces cell damage, leading to cell death via different pathways.

The pathophysiology of IRI can be segregated into distinct phases: ischemia and reperfusion [18]. During ischemia, compromised oxygen delivery forces cells to switch to anaerobic metabolism, leading to mitochondrial dysfunction and diminished ATP production. This subsequent ATP depletion impairs the function of Na^+^/K^+^-ATPase, resulting in an influx of Ca^2+^, H^+^, and Na^+^ ions. This ionic dysregulation triggers cellular edema and disrupts cytoplasmic enzyme activity. The Ca^2+^ overload directly contributes to cellular injury through various mechanisms, including the disruption of cell membranes, induction of apoptosis, and further impairment of mitochondrial function (Figure 1) [19].

Reperfusion paradoxically exacerbates tissue damage. Mitochondrial dysfunction and electrolyte imbalances during this phase culminate in the rapid and substantial generation of ROS from multiple sources within minutes. These sources include upregulated enzymes like xanthine oxidase (XO), nicotinamide adenine dinucleotide phosphate oxidase (NOX), cyclooxygenase (COX), lipoxygenase (LOX), and inducible nitric oxide synthase (iNOS), alongside mitochondrial electron transport chain reactions and catecholamine oxidation [20]. The ROS produced overwhelm cellular antioxidant defenses, leading to the destruction of cell and organelle membranes, DNA breakage, and subsequent enzyme inactivation. This culminates in cell death through various pathways, including necrosis, apoptosis, autophagy, mitoptosis, and necroptosis (Figure 1) [21].

Hypoxia induced during ischemic injury triggers a systemic inflammatory response characterized by the production of pro-inflammatory cytokines and adhesion molecules, e.g., tumor necrosis factor-alpha (TNF-α), interleukin-1 (IL-1), interleukin-6 (IL-6), interleukin-8 (IL-8), and platelet-activating factor (PAF). This cytokine surge is further amplified by the activation of the transcription factor nuclear factor-kappa B (NF-κB) [1,22]. The activation of NF-κB occurs during both the ischemic and reperfusion phases [23]. Reperfusion then activates the mobilization and infiltration of neutrophils, which generate ROS and pro-inflammatory cytokines such as TNF-α. These mediators collectively exacerbate tissue injury [24].

The endothelium is important organ that participates in inflammation. Ischemic insults, including hypoxia and hypoglycemia, trigger a signaling cascade that upregulates the expression of endothelial adhesion molecules, such as intercellular adhesion molecule-1 (ICAM-1) and vascular cell adhesion molecule-1 (VCAM-1). During reperfusion, the coordinated upregulation of these endothelial adhesion molecules facilitates the adhesion, transmigration, and extravasation of neutrophils to the IRI site. The recruited neutrophils then release ROS and a plethora of pro-inflammatory mediators, further amplifying the inflammatory response and tissue damage [25].

Compelling evidence from numerous studies has demonstrated that oxidative stress plays a critical role in the pathogenesis of reperfusion injury. Re-oxygenation during reperfusion increases the amounts of ROS that destabilize important biomolecules, leading to cell injury [1,26]. Reperfusion injury is characterized by the enhancement of ROS generation through multiple mechanisms, including upregulated XO activity, neutrophil activation, and the dysfunction of the mitochondrial electron transport chain [27]. These ROS species exert cytotoxic effects via lipid peroxidation or protein and DNA oxidation [28]. ROS initiate a chain reaction, leading to the peroxidation of polyunsaturated fatty acids within cell membranes. This disrupts membrane integrity and generates deleterious secondary metabolites like malondialdehyde (MDA) [29]. ROS and reactive nitrogen species (RNS), such as peroxynitrite, directly modify proteins and DNA. This modification can inactivate critical enzymes like superoxide dismutase (SOD) through tyrosine nitration and disrupt DNA integrity [30]. In addition, oxidative reactions may damage nucleic acids and chromosomes during IRI [31]. Clinical evidence supports this, as elevated plasma levels of the oxidative DNA marker 8-hydroxy-2′-deoxyguanosine (8-OHdG) have been observed in adult patients with myocardial infarction undergoing reperfusion [32].

Cell fate after IRI largely depends on its duration, as well as on the extent of induced damage. A shorter duration of IRI may activate cell survival programs to control ROS production and cell damage [33]. Moderate IRI may cause cell dysfunction by autophagy and activate recovery systems for survival. If damage is severe enough, cell death may be induced via apoptotic or necrotic pathways [34]. The extrinsic apoptotic pathway, also known as the death receptor pathway, is activated by death ligands and receptors, including TNF-α, the Fas ligand, or the TNF-related apoptosis inducing ligand (TRAIL). This activates caspase-8 to cleave caspase-3, which then induces cell death via proteolysis in damaged cells. The intrinsic pathway, also known as the mitochondrial pathway, is activated, e.g., by hypoxia, leading to the activation of the pro-apoptotic B-cell lymphoma 2 (Bcl-2) family, release of cytochrome c, and activation of caspase-9, thereby driving apoptosis [2]. The main characters of necrosis are cell disintegration, organ swelling, and loss of mitochondrial function. Necrosis induces a large number of local inflammatory responses in ischemic tissue [35]. The surge in ROS production, predominantly observed during reperfusion, triggers the opening of the mitochondrial permeability transition pore (mPTP). This event dissipates the mitochondrial membrane potential, compromising the proton gradient essential for ATP production. The influx of water and solutes into the mitochondrial matrix due to the open mPTP leads to mitochondrial swelling. The disruption of the outer mitochondrial membrane by the mPTP opening allows the release of pro-apoptotic molecules, such as cytochrome c, into the cytosol. Cytochrome c release in the cytosol initiates a cascade of events, culminating in the activation of the intrinsic apoptotic pathway [36].

Ischemic insult, characterized by oxygen depletion, compromises the electron transport chain within the mitochondrial respiratory chain. This impairment disrupts oxidative phosphorylation, leading to a subsequent decrease in ATP levels. This condition leads to the failure of the sodium–potassium pump, which causes the retention of sodium in cells and flow of potassium out of cells [37]. In addition, anaerobic glycolysis induced by hypoxia generates increased cytosolic lactate, which lowers intracellular pH. Increased H^+^ activates the Na^+^/H^+^ exchanger, which further worsens the cytosolic Na^+^ overload. In response to the cytosolic Na^+^ overload, the cell activates the Na^+^/Ca^2+^ exchanger in its reverse mode, which shifts intracellular Na^+^ outside the cells while introducing Ca^2+^ into the cells, thereby causing calcium overload [38]. Calcium pumps on the endoplasmic reticulum also become dysfunctional, which limits calcium re-uptake [39]. Reperfusion results in the opening of mPTP. The intracellular increase in Ca^2+^ enhances its influx into the mitochondria, causing the loss of the impermeability of the internal mitochondrial membrane, thereby leading to mitochondrial dysfunction and apoptosis [40].

## 3. Protective Effects of Molecular Hydrogen on Ischemia/Reperfusion Injury

**Figure 2:**
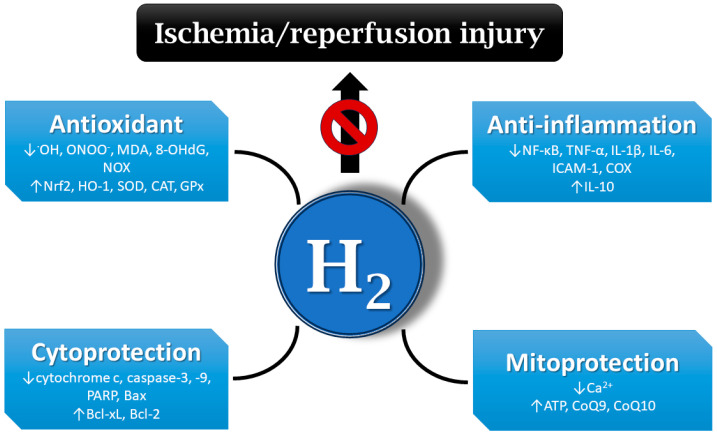
Mechanisms of hydrogen’s protective effects on ischemia/reperfusion injury (IRI). Hydrogen may be effective at mitigating IRI via different mechanisms, e.g., antioxidant and anti-inflammatory action, the regulation of cell death, balancing ion homeostasis, the enhancement of ATP production, etc. ^•^OH—hydroxyl radical; ONOO^−^—peroxynitrite; MDA—malondialdehyde; 8-OHdG—8-hydroxy-2′-deoxyguanosine; NOX—nicotinamide adenine dinucleotide phosphate oxidase; Nrf2—nuclear factor erythroid 2-related factor 2; HO-1—heme oxygenase 1; SOD—superoxide dismutase; CAT—catalase; GPx—glutathione peroxidase; NF-κB—nuclear factor kappa B; TNF-α—tumor necrosis factor alpha; IL-1β—interleukin 1 beta; ICAM-1—intercellular adhesion molecule 1; COX—cyclooxygenase; PARP—poly (ADP-ribose) polymerase; Bax—Bcl-2-associated X-protein; Bcl-xL—B-cell lymphoma-extra-large; Bcl-2—B-cell lymphoma 2; ATP—adenosine triphosphate; CoQ9—coenzyme Q9; H_2_—hydrogen.

A number of publications document that molecular hydrogen could play an important role in the prevention and treatment of IRI [10,11,12,41]. IRI-related protective effects of hydrogen are mediated via different mechanisms, including the inhibition of inflammatory cytokine secretion and reduction in oxidative stress, balancing intracellular ion homeostasis, mitigating mitochondrial damage, and regulating cell death and other signaling pathways [42] (Figure 2).

It has been demonstrated that molecular hydrogen can directly and selectively scavenge the strong oxidants ^•^OH and ONOO^−^ in a model of cerebral IRI, whereas other ROS acting as signaling molecules were not compromised [4]. Hydrogen administration also decreased the formation of oxidative stress markers, such as MDA or 8-OHdG [43,44]. Other studies reported that hydrogen exerts antioxidant activity via the activation of the nuclear factor erythroid 2-related factor 2 (Nrf2) pathway, thereby regulating its downstream antioxidant molecules [45]. In an ischemia model, hydrogen reduced the production of ^•^OH, promoted Nrf2 nuclear translocation, and regulated the Nrf2/HO-1 pathway in H9c2 cells [46]. In rats, hydrogen-rich water can also upregulate the expression of other antioxidant enzymes, including SOD, catalase (CAT), and glutathione peroxidase (GPx) [47,48,49]. On the other hand, hydrogen showed inhibitory effects on ROS-producing enzymes, such as NOX in hypertensive rats [49,50].

In one of the early studies, the inhalation of molecular hydrogen in hyperbaric chamber showed anti-inflammatory effects in a mouse model of parasite-induced liver inflammation [51]. The anti-inflammatory effect of molecular hydrogen may involve the inhibition of several inflammatory pathways, including the NF-κB pathway [52]. Hydrogen also displayed an anti-inflammatory effect in a model of cerebral IRI by upregulating regulatory T cells, which was accompanied with the inhibition of NF-κB and TNF-α expression [53]. Hydrogen inhalation ameliorated intestinal transplant injury by lowering the inflammatory mediators chemokine ligand 2 (CCL-2), IL-1β, IL-6, and TNF-α [54]. At the same time, hydrogen increases the expression of the anti-inflammatory factor IL-10 [55]. The inhibition of the release of adhesion molecules, e.g., ICAM-1, by molecular hydrogen has also been demonstrated [56]. Available information points to the ability of hydrogen to inhibit inflammatory enzymes, such as COX [57].

In most publications, molecular hydrogen exerted anti-apoptotic effects by modulating the expression of apoptosis-associated proteins. In radiation models built both in mice and in an intestinal crypt epithelial cell (IEC-6) line, hydrogen attenuates radiation-induced intestinal damage and prevents cytochrome c release and activity of caspase-3, caspase-9, and poly (ADP-ribose) polymerase (PARP). Moreover, the expressions of anti-apoptotic B-cell lymphoma-extra-large (Bcl-xL) and Bcl-2 proteins were increased, whereas the expression of pro-apoptotic Bcl-2-associated X-protein (Bax) was decreased after hydrogen treatment [58]. Similarly, hydrogen inhibited the levels of pro-apoptotic molecules caspase-3 and caspase-8 in rat lung grafts, elevated the expression of anti-apoptotic molecules Bcl-2 and Bcl-xL, and stabilized the mitochondrial outer membrane, preventing the release of cytochrome c into the cytosol [59]. Hydrogen can also activate the mitogen-activated protein kinase/heme oxygenase 1 (MAPK/HO-1) pathway to inhibit neuronal apoptosis and alleviate ischemic brain injury in neonatal mice [60]. Furthermore, in rat IRI, molecular hydrogen can inhibit apoptosis by reducing inflammation and oxidative damage but activating autophagy [61]. By activating the autophagy pathway, molecular hydrogen alleviated endoplasmic reticulum stress and mitigated inflammation and organ injury in mice [62].

Several studies demonstrated the calcium-lowering effects of molecular hydrogen in different experimental settings. In one of them, the administration of hydrogen-rich water protected against ischemic brain injury, where hydrogen-saturated water attenuated the elevation of intracellular Ca^2+^ levels [13]. Another study reported that hydrogen inhibits oxidative stress-induced MAPK activation and maintains calcium homeostasis in airway epithelial cells [63]. The ability of hydrogen to regulate calcium levels might be effective at attenuating IRI.

Hydrogen also exerts its protective effects on IRI via the mitigation of mitochondrial damage. The inhalation of hydrogen improved mitochondria function through increased mitochondrial membrane potential and ATP levels and promoted the activity of mitochondrial–respiration complexes in acute lung injury of mice [64]. In an acute myocardial infarction model, hydrogen gas activated mitochondrial ATP-sensitive K^+^ channels (mKATP) and regulated mitochondrial membrane potential and the production of mitochondrial ATP, thus alleviating myocardial IRI in dogs [65]. Hydrogen application significantly increased CoQ9 concentrations in the plasma and myocardium tissue of rats. Increased CoQ9 levels improved ATP production via mitochondrial oxidative phosphorylation in rats [66]. Hydrogen activated CoQ10 in the mitochondria of oncological patients treated with nivolumab, thereby enhancing its efficacy [67].

### 3.1. Cardiovascular System

Preclinical investigations utilizing animal models indicate that myocardial IRI contributes substantially to the final infarct size, potentially accounting for up to 50% of the tissue injury observed in myocardial infarction [68]. Hayashida et al. [69] demonstrated that the inhalation of hydrogen gas has a cardioprotective effect by reducing the infarct size in a rat model of myocardial IRI. In another study, the authors reported that isolated rat heart hydrogen-saturated Krebs–Henseleit solution significantly decreased infarct size induced by myocardial ischemia/reperfusion (I/R) hypoxic post-conditioning [70]. In a rat model of IRI, hydrogen inhalation led to significant improvements in myocardial infarct size, cardiac function, microstructure, and mitochondrial morphology. Additionally, the levels of 8-OHdG, MDA, ROS, and pyroptosis-related proteins were markedly reduced [12]. Hydrogen-rich water can activate the phosphoinositide 3-kinase/protein kinase B (PI3K/AKT) signaling pathway, alleviate IRI in isolated rat hearts, and inhibit cardiomyocyte apoptosis [71]. Sun et al. [72] also showed that hydrogen could reduce myocardial damage in a rat heart with regional myocardial I/R through antioxidative and anti-inflammatory effects.

The use of hydrogen in heart transplantation seems to be associated with reduced oxidative stress and inflammation [42]. Nakao et al. [73] showed that hydrogen could significantly reduce heart IRI induced by prolonged hypothermic preservation prior to transplantation, as revealed by decreased levels of MDA, troponin I, and creatine phosphokinase. Similarly, Noda et al. [74] documented that a hydrogen-supplemented preservation solution efficiently improved myocardial injury due to cold I/R in a rat transplantation model by decreasing the levels of various inflammatory markers (IL-6, IL-1β, TNF-α, ICAM-1, iNOS, and CCL2). Another study by Noda et al. [75] showed that drinking hydrogen-rich water after heart transplantation led to the elimination of toxic ROS, increased ATP levels, and enhanced mitochondrial respiratory chain function. At the same time, levels of IL-2 and interferon gamma (IFN-γ) were reduced, along with the mitigation of intimal hyperplasia.

### 3.2. Respiratory System

I/R-induced lung injury usually occurs after cardiac bypass surgery and lung transplantation, leading to primary graft dysfunction [28]. Studies on the application of molecular hydrogen in I/R-induced lung injury are focused on lung transplantation models, where molecular hydrogen reduced oxidative stress, inflammation, and apoptosis, thus alleviating I/R lung injury [52].

The pre-treatment of lungs with hydrogen-rich saline via lung immersion facilitated hydrogen delivery to the pulmonary tissue, resulting in the attenuation of lung IRI [76]. A rat lung transplantation model demonstrated that employing a hydrogen-rich preservation solution during cold ischemia attenuated lung IRI, likely through its antioxidant and anti-inflammatory properties [41]. A study by Liu et al. [59] showed that lung inflation with hydrogen during the cold ischemia phase lowered graft myeloperoxidase (MPO) activity and serum IL-8 and TNF-α levels, resulting in decreased lung graft injury. A study in pigs showed that hydrogen gas inhalation improved lung function after donation following cardiac death. The hydrogen group revealed lower expression of IL-1β, IL-6, IL-8, and TNF-α, as well as lower scores for lung injury severity [77].

Molecular hydrogen could also inhibit apoptosis in rat lung transplantation via reduced expression of pro-apoptotic caspase-3 and caspase-8 in lung grafts and by increasing the anti-apoptotic proteins Bcl-2 and Bcl-xL, thus stabilizing the mitochondrial outer membrane and terminating the release of cytochrome c into the cytosol. In another study, the inhalation of hydrogen alleviated lung graft IRI by reducing inflammatory mediators, lowering tissue MDA levels, and increasing the levels of anti-apoptotic Bcl-2 and Bcl-xL proteins [78].

Molecular hydrogen demonstrated a protective effect against lung injury induced by limb I/R. Specifically, hydrogen reduced MDA levels and enhanced SOD activity in lung tissues. Additionally, hydrogen activated Nrf2 signaling and exhibited the ability to inhibit the upregulation of autophagy in the present rodent model [79].

### 3.3. Kidneys

Renal IRI may be induced by multiple conditions, such as renal transplantation [80] or sepsis [81]. Hydrogen-rich saline used during the hypothermic preservation of renal grafts has been shown to decrease oxidative stress (MDA and 8-OHdG levels) and prolong graft survival [82]. Similarly, Shingu et al. [83] demonstrated that treatment with hydrogen-rich saline could significantly alleviate renal graft IRI by reducing the levels of 8-OHdG, therefore improving renal transplant function and maintaining normal tissue structure after transplantation. In another study, oral administration of hydrogen-rich saline improved overall survival after kidney transplantation through the reduction in oxidative stress and limiting inflammation [84].

Renal IRI was induced in an experimental model by subjecting the bilateral renal pedicles to 45 min of ischemia, followed by a reperfusion period of 108 h. Hydrogen-rich saline injected intraperitoneally at 4 h intervals attenuated renal IRI through reductions in inflammation (TNF-α, IL-6) and apoptosis (Bcl-2, caspase-3, -8, -9) [85]. In an analogous experimental model, hydrogen-rich saline enhanced the renal response to IRI in aged rats. This effect is likely mediated by the reduction in oxidative stress and the upregulation of HO-1 gene expression [86].

### 3.4. Liver

Hepatic IRI is caused by pathological and surgical factors, such as liver resections [87] or its transplantations [88]. In an experimental pig model, the inhalation of hydrogen gas during major liver resection demonstrated a protective effect by attenuating oxidative stress levels associated with IRI [11]. Matsuno et al. [89] examined the perfusion of the donor liver with hydrogen-saturated lactate Ringer’s solution just before reperfusion. The results of the study showed significantly lower aspartate aminotransferase and lactate dehydrogenase levels in animals with hydrogen-perfused livers, suggesting better graft function than in untreated grafts. In a liver transplantation model, hydrogen gas inhalation protected against liver IRI by activating the NF-κB signaling pathway [90]. The storage of liver grafts in hydrogen-rich solution protected these grafts against IRI via the up-regulation of HO-1 expression [91].

In a mouse model of fatty liver, hydrogen treatment exerted significant suppression of IRI. This effect was achieved by reducing hepatocyte apoptosis, inhibiting macrophage activation and inflammatory cytokines, and inducing the expression of HO-1 and Sirtuin1 (Sirt1) [10]. Similarly, in a mouse model of liver IRI, Fukuda et al. [92] found that hydrogen inhalation could significantly reduce liver IRI by inhibiting the release of serum alanine aminotransferase and MDA production.

### 3.5. Brain

Ischemic stroke, cardiac arrest, trauma, and perinatal hypoxic ischemic injury are common causes of brain IRI [93]. Hydrogen has been shown to mitigate acute spinal cord injury by enhancing the release of brain-derived neurotrophic factor (BDNF) and reducing levels of oxidative products, including 8-iso-prostaglandin F2α and MDA [94]. In a study by Kimura et al. [95], hydrogen gas inhalation demonstrated a protective effect against spinal ischemic injury. In mice with focal cerebral IRI, molecular hydrogen significantly increased SOD and GPx activity, reduced MDA levels and infarct volume, relieved cerebral oedema, improved neurological outcomes, and alleviated cognitive deficits [96].

Hydrogen has been shown to protect against oxidative stress and neuroinflammation in rats with local cerebral ischemia [97]. Hydrogen treatment of mice after bilateral carotid artery occlusion improved cognitive abilities and induced anti-apoptotic and antioxidant effects [98]. Rats given hydrogen-rich water before and after the occlusion of the middle cerebral artery showed reduced infarct volumes and improved neurological outcomes [13].

Hydrogen inhalation for 4 days improved neurological outcomes and survival after global cerebral ischemia due to cardiac arrest in systemic hypertension rats [99]. The intraperitoneal injection of hydrogen into rabbits in cardiac arrest improved 3-day survival and neurological deficits, reduced neuronal damage, and inhibited neuronal apoptosis [100].

## 4. Clinical Applications

It is well known that the administration of antioxidants has a positive effect on various diseases in animal and in vitro studies. However, their effect is not often observed in humans. Since the administration of hydrogen has had mostly positive results in the treatment of many diseases in animals and in vitro experiments, the question is whether such effects will also be manifested in human studies. Currently, there are more than 80 identified clinical trials and more than 64 scientific publications related to hydrogen therapy in human studies. These trials cover major disease areas, including cardiovascular diseases, cancer, respiratory diseases, central nervous system disorders, and infections [101].

Sim et al. [102] observed that in healthy adults, 4 weeks of administration of hydrogen-rich water significantly increased biological antioxidant potential and decreased peripheral blood mononuclear cells apoptosis and levels of cluster of differentiation 14+ (CD14+). These findings suggest that hydrogen administration may enhance antioxidant capacity, potentially leading to reductions in inflammatory responses in healthy adults. Further investigation is warranted to elucidate the underlying mechanisms and confirm these observations in a clinical setting. In the study of Takeuchi et al. [103], hydrogen administration exerts encouraging effects in patients with delayed cerebral ischemia and vasospasms induced by aneurysmal subarachnoid hemorrhage. The authors observed decreasing values of MDA and neuron-specific enolase. In connection to IRI, it was found that hydrogen inhalation treatment significantly reduced levels of oxidative stress and inflammation markers in patients with post-cardiac arrest syndrome [104]. Health-promoting effects of hydrogen therapy were also observed in patients with adverse left ventricular remodeling after percutaneous coronary intervention after myocardial infarction. A 6-month treatment with 1.3% hydrogen inhalation improved the left ventricular stroke volume index and ejection fraction [105].

Hydrogen administration also seems to be an effective treatment in oncological diseases. In patients suffering from recurrent gallbladder carcinoma, the administration of hydrogen inhalation therapy led to a decrease in tumor size, and tumor marker levels returned to normal values [106]. In another study, Kang et al. [107] observed that patients with liver tumors treated with radiotherapy and with hydrogen-rich water had reduced parameters of oxidative stress and improved quality of life scores. Interestingly, hydrogen treatment had no effect on the efficacy of radiotherapy to the tumor.

In humans, the positive effect of molecular hydrogen administration has also been observed in metabolic diseases. Korovljev et al. [108] proved that treatment with molecular hydrogen-rich water significantly reduced body fat, the arm fat index, serum triglycerides, and serum insulin levels in middle-aged overweight women. In a randomized, double-blinded, placebo-controlled trial involving subjects with metabolic syndrome, supplementation with high-concentration hydrogen-rich water led to several beneficial effects. Hydrogen-rich water significantly reduced blood cholesterol and glucose levels, attenuated serum hemoglobin A1c, and improved biomarkers related to inflammation and redox homeostasis. Additionally, there was a tendency for hydrogen-rich water to promote a mild reduction in body mass index (BMI) and waist-to-hip ratio [109]. A beneficial effect of hydrogen treatment was observed in patients with non-alcoholic fatty liver disease. Patients drinking hydrogen-rich water had improved lipid profiles, reduced lactate dehydrogenase levels, and decreased levels of NF-κB, heat shock protein 70, and matrix metalloproteinase-9 after eight weeks treatment [110].

The administration of hydrogen could also be used in regenerative medicine to speed up recovery after injury. It was observed that oral and topical hydrogen intervention is potentially effective in the treatment of soft tissue injuries in male professional athletes [111]. Significant results pointing to the beneficial effect of hydrogen in sport medicine research were achieved by Botek et al. [112]. This randomized, double-blinded, placebo controlled, crossover study revealed that pre-exercise consumption of hydrogen-rich water leads to a significant improvement in sprint times, ranging from 1.9% to 3.4%. However, the authors did not prove that drinking hydrogen-rich water decreases lactate concentrations or ratings of perceived exertion but concluded that pre-exercise hydrogen-rich water supplementation is associated with an increased ability to reduce fatigue, especially during the later stages of repeated sprint exercise. In another study, hydrogen inhalation demonstrated favorable health effects, resulting in improvements in the 6 min walking distance test, forced vital lung capacity, and expiratory volume in the first second in acute post-COVID-19 patients [113].

It is obvious that interest in hydrogen therapy continues to grow, and it may become a new drug substance in future clinical practice.

## 5. Future Research Directions

In the future, scientists should explore the mechanisms of hydrogen’s protective effects and elucidate the specific molecular pathways and signaling cascades through which hydrogen mediates its protective effects, such as the roles of Nrf2, HO-1, Sirt1, and other key regulators. It is essential to evaluate hydrogen in clinically relevant transplantation models and examine the protective effects of hydrogen in more clinically relevant models of organ transplantation, which involve prolonged cold ischemia, followed by reperfusion, to validate the translational potential of hydrogen therapy.

It will be necessary to optimize hydrogen delivery methods and systematically compare the efficacy and feasibility of different hydrogen delivery approaches, such as the inhalation of hydrogen gas, the administration of hydrogen-rich saline, or drinking hydrogen-rich water, to determine the optimal method for clinical applications. It is also necessary to investigate combination therapies and explore the potential of combining hydrogen therapy with other pharmacological or therapeutic interventions to provide additive or synergistic protective effects, which may lead to more effective treatments. It would also be useful to expand to other ischemic conditions and investigate the protective effects of hydrogen in a broader range of ischemic conditions, such as myocardial infarction, stroke, and acute kidney injury, to further demonstrate the therapeutic potential of hydrogen.

By addressing these research directions, future studies can build upon the existing knowledge, elucidate the mechanisms, optimize the delivery, and establish the clinical relevance of molecular hydrogen as a promising therapeutic intervention for ischemia/reperfusion injury.

## 6. Conclusions

Ischemia/reperfusion injury still remains an important clinical problem in medicine. The overproduction of free radicals, the release of pro-inflammatory cytokines, ionic imbalance, and mitochondrial disruption are concomitant problems of ischemia/reperfusion injury leading to cell death and tissue injury, and it is a serious complication for patients. Numerous studies are now focusing on the benefits of using free radical scavengers, such as antioxidants, to mitigate the detrimental impacts of excessive free radicals while simultaneously restoring blood and oxygen supply to ischemic tissues. Molecular hydrogen appears to be one such substance, especially based on very promising experiments on cells and animal models of various diseases. Extensive experimental evidence indicates that hydrogen can effectively mitigate ischemia/reperfusion injury associated with transplantation and has therapeutic benefits for transplantation-related complications. These effects primarily result from inhibiting inflammatory cytokine secretion and reducing oxidative stress. However, the precise underlying mechanisms remain poorly understood, and their clarification will require deeper research either using animal models or human clinical studies.
